# Influence of oral glutamine supplementation on survival outcomes of patients treated with concurrent chemoradiotherapy for locally advanced non-small cell lung cancer

**DOI:** 10.1186/1471-2407-12-502

**Published:** 2012-10-31

**Authors:** Erkan Topkan, Cem Parlak, Savas Topuk, Berrin Pehlivan

**Affiliations:** 1 Department of Radiation Oncology, Baskent University Adana Medical Faculty, Adana, Turkey; 2 Department of Radiation Oncology, Memorial Health Group, Medstar Antalya Hospital, Antalya, Turkey

**Keywords:** Concurrent chemotherapy, Radiotherapy, Glutamine supplementation, Lung cancer, Survival outcome, Tumor growth

## Abstract

**Background:**

Glutamine (Gln) supplementation during concurrent chemoradiotherapy (C-CRT) effectively reduces the incidence and severity of acute radiation-induced esophagitis (RIE). However, there are concerns that Gln might stimulate tumor growth, and therefore negatively impact the outcomes of anticancer treatment. We retrospectively investigated the effect of co-administration of oral Gln during C-CRT on survival outcomes of patients with stage IIIB non-small cell lung carcinoma (NSCLC). We additionally evaluated role of oral Gln in preventing C-CRT-induced weight change, acute and late toxicities.

**Methods:**

The study included 104 patients: 56 (53.8%) received prophylactic powdered Gln (Gln+) orally at a dose of 10 g/8 h and 48 (46.2%) did not receive Gln (Gln-) and served as controls. The prescribed radiation dose to the planning target volume was 66 Gy in 2-Gy fractions. Primary endpoints of progression-free survival (PFS), local/regional progression-free survival (LRPFS), and overall survival (OS) were correlated with status of Gln supplementation.

**Results:**

Oral Gln was well tolerated except for mild nausea/vomiting in 14 (25.0%) patients. There was no C-CRT-related acute or late grade 4–5 toxicity. Administration of Gln was associated with a decrease in the incidence of grade 3 acute radiation-induced esophagitis (RIE) (7.2% vs. 16.7% for Gln+ vs. Gln-; p=0.02) and late-RIE (0% vs. 6.3%; p=0.06), a reduced need for unplanned treatment breaks (7.1% vs. 20.8%; p=0.04), and reduced incidence of weight loss (44.6% vs. 72.9%; p=0.002). At a median follow-up of 24.2 months (range 9.2-34.4) the median OS, LRPFS, and PFS for Gln+ vs. Gln- cohorts were 21.4 vs. 20.4 (p=0.35), 14.2 vs.11.3 (p=0.16), and 10.2 vs. 9.0 months (p=0.11), respectively.

**Conclusion:**

In our study, supplementation with Gln during C-CRT had no detectable negative impact on tumor control and survival outcomes in patients with Stage IIIB NSCLC. Furthermore, Gln appeared to have a beneficial effect with respect to prevention of weight loss and unplanned treatment delays, and reduced the severity and incidence of acute- and late-RIE.

## Background

Complications related to concurrent chemoradiotherapy (C-CRT) such as acute radiation-induced esophagitis (ARIE) may cause significant morbidity and unplanned treatment delays in patients with locally advanced non-small cell lung carcinoma (LA-NSCLC). Such complications not only impact the quality of life but also reduce the ability to escalate the dose of radiotherapy (RT) to more effective levels, resulting in potential reductions in tumor control and survival rates. Improvements in target definition and the advent of sophisticated RT techniques, combined with elimination of elective irradiation of clinically uninvolved lymphatics, have significantly reduced the volume of normal tissue exposed to high-dose radiation with a resultant reduction in incidence and severity of treatment-related toxicity [1]. However, because of the need to irradiate subclinical tumor extension, normal tissue toxicity and its consequences likely will remain a challenge for the foreseeable future [2].

Pharmacologic radioprotection can efficiently prevent, or at least reduce, the incidence and/or severity of acute radiation-induced esophagitis (ARIE) and related complications during C-CRT of LA-NSCLC. One agent with potential radioprotective properties is glutamine (Gln), the primary oxidative fuel of the gut epithelium that is necessary for maintenance of its structural integrity [3,4]. Although Gln is continuously provided by skeletal muscles during hypercatabolic states such as cancer, over time marked Gln depletion develops that cannot be overcome by increased synthesis [4]. This results in compromised acid–base balance, immune functions, and epithelial integrity in the gut [5]. Additionally, because of its antioxidant activity in normal tissues, depletion of glutathione (GSH), a by-product of Gln metabolism, may increase the extent of tissue damage caused by C-CRT [3,6,7]. In this context, exogenous Gln supplementation not only normalizes Gln levels in the body but also selectively increases GSH levels in normal tissue, which may explain its selective radioprotective function [3,6-8]. Two recent studies, including one from our institution, revealed a beneficial role of oral Gln in the reduction of ARIE incidence and severity, as well as maintenance of body weight, in LA-NSCLC patients treated with C-CRT [9,10].

It is important to investigate the effect of any agent that reduces treatment-related toxicities on tumor tissue. As an example, amifostine, which is a strong radioprotector, was found to have no detrimental effects on survival outcome in a recent meta-analysis by Bourhis et al. [11], suggesting no tumor protection or growth stimulating action. On the contrary, erythropoietin, which has been used successfully for stimulation of erythropoiesis in various cancers, negatively impacted survival outcomes for most tumor types [12]. Considering these two conflicting results of two agents, commonly practiced in radiation oncology clinics, because growth of various cell lines of tumor and non-tumor origin is a function of Gln availability [13-15], there is increasing concern that Gln might stimulate tumor growth and therefore negatively impact outcomes of anticancer treatment. This issue has never been addressed in the setting of NSCLC. Therefore, in this retrospective analysis, we comparatively assessed the impact of Gln supplementation during C-CRT on survival outcomes in LA-NSCLC patients. We additionally evaluated role of oral Gln in preventing C-CRT-induced weight change, acute and late toxicities.

## Methods

### Study subjects

The database maintained by our institution was retrospectively searched to identify all patients with LA-NSCLC who had undergone C-CRT between January 2008 and December 2010. Inclusion criteria were: histopathologically proven NSCLC, stage IIIB disease by 18F-fluorodeoxyglucose positron emission tomography (FDG PET-CT), age ≥18 and <70, Karnofsky Performance Status (KPS) ≥70, available treatment charts and hospital computerized data, RT data sets for dosimetric calculations, no prior history of thoracic RT (TRT) or chemotherapy, no contraindication for C-CRT, no pre-treatment dysphagia or ingestion difficulties, body mass index (BMI) ≥18 kg/m^2^, and no dietary supplementation except for Gln in the prescribed dose and schedule. The study population contained 104 patients who met the above criteria.

The study was approved by the institutional review board of Baskent University before collection of patient information and was conducted according to the principles of the Declaration of Helsinki and the rules of Good Clinical Practice.

### Concurrent chemoradiotherapy

In our department, FDG-PET-CT fusion-based three-dimensional treatment planning is the standard of care for LA-NSCLC patients. Target volume definition, dose specification, and normal tissue tolerance limits for eligible patients were as described elsewhere [10]. Briefly, TRT was administered through anteroposterior-posteroanterior (AP-PA) portals with individualized multi-leaf collimator blocks for initial planning target volume (PTV1) up to 46 Gy, followed by an off-spinal cord oblique boost dose of up to 66 Gy for PTV2. All patients received daily TRT for 5 days a week with 2 Gy per fraction using high energy linear accelerators and concurrent treatment with one of the two following chemotherapy combinations: CD, cisplatin (80 mg/m^2^) and docetaxel (80 mg/m^2^), on days 1, 22, and 43 (n=49); or CV, cisplatin (80 mg/m^2^) and vinorelbine (30 mg/m^2^, days 1 and 8) every 21 days for 3 cycles (n=55).

### Glutamine supplementation

Our current institutional policy is to recommend prophylactic Gln supplementation for all patients scheduled to undergo TRT. We prefer to use oral Gln powder to reduce the incidence and severity of ARIE. Fifty-six patients (53.8%) received powdered Gln (Nestle Nutrition, Istanbul, Turkey) at a dose of 10 g/8 h orally in water or fruit juice, starting 1 week before TRT and continuing for 2 weeks after completion of RT. The remaining 48 patients (46.2%), who did not receive Gln due to economic reasons or patients’ self-choice, served as controls. Based on institutional standards, patients receiving Gln were followed by experienced nurses for adherence to protocol, general nutritional status, and adverse events throughout the treatment period. The dose of 30 g/day was selected based on available literature, which reported its efficacy in reducing the incidence and severity of ARIE and weight loss in LA-NSCLC patients treated with C-CRT [9,10] and in lowering the incidence of grade 2–4 mucositis in patients treated with cytotoxic chemotherapy [16,17]. Patients who did not use Gln were nourished with diets that were achievable based on their socioeconomic status to improve their nutritional status.

### Patient evaluation and toxicity scoring

For each patient, we calculated weight change (WC), percent WC (PWC), and body mass index (BMI) change between baseline and post-treatment measures using available chart records. Weight change, the absolute difference between pre- and post-treatment weight measures, is a parameter that is independent of pre-treatment weight and has the potential to underestimate the value of pre-treatment body mass [18]. Therefore, we also calculated weight change as a percentage relative to pre-treatment weight (PWC). Nausea and vomiting was considered Gln-induced only if reported within the 1-week period of Gln administration before commencement of C-CRT, and graded according to RTOG scoring [19]. All patients were examined at weekly intervals for ARIE incidence and weight changes during C-CRT. ARIE was graded by a radiation oncologist according to RTOG-ARIE scoring criteria [19], and the reported grade of ARIE reflected the worst grade observed (Table 1). The calculated and reported data were used for intra- and intergroup comparisons. After completion of C-CRT, patients were examined at weekly intervals for the first month to allow for the possibility of an early “esophagitis peak” and bimonthly thereafter.

**Table 1:** Radiation Therapy Oncology Group (RTOG) acute radiation-induced esophageal morbidity scoring criteria

**Grade**	**Description**
0	No change
1	Mild dysphagia or odynophagia, requiring topical anesthetic, non-narcotic agents, or soft diet
2	Moderate dysphagia or odynophagia, requiring narcotic agents or liquid diet
3	Severe dysphagia or odynophagia with dehydration or weight loss (>15% of pretreatment baseline), requiring nasogastric feeding
4	Complete stricture, ulceration, perforation or fistula
5	Death

### Response assessment and follow-up

Treatment response was assessed by re-staging FDG-PET-CT scans from the 8-week post-C-CRT follow-up according to EORTC-1999 guidelines [20] (summarized in Table 2), and at 3-month intervals thereafter. The 8-week time interval for the first follow-up FDG-PET-CT was arbitrarily chosen as the shortest possible time for response assessment based on our national health insurance politics, rather than on evidence-based practice. Thereafter, patients were monitored by evaluation of blood count/chemistry every 8–12 weeks. Additional abdominal ultrasound and/or CT, chest CT, cranial magnetic resonance imaging, and FDG-PET-CT were performed as indicated.

**Table 2:** Proposed EORTC 1999 criteria for clinical and subclinical response assessment by PET-CT

**Response**	**Definition**
**Progressive metabolic disease**	An increase in ^18^FDG tumor SUV of greater than 25% within the tumor region defined on the baseline scan, visible increase in the extent of ^18^FDG tumor uptake (>20% in the longest dimension) or the appearance of new ^18^FDG uptake in metastatic lesions
**Stable metabolic disease**	An increase in tumor ^18^FDG SUV of less than 25% or a decrease of less than 15% and no visible increase in extent of ^18^FDG tumor uptake (>20% in the longest dimension)
**Partial metabolic response**	A reduction of a minimum of 15–25% in tumor ^18^FDG SUV after one cycle of chemotherapy, and greater than 25% after more than one treatment cycle
**Complete metabolic response**	Complete resolution of ^18^FDG uptake within the tumor volume so that it was indistinguishable from surrounding normal tissue

### Statistical methods

Statistical analyses were performed based on patient stratification according to their Gln supplementation status (Gln+ and Gln-). Frequency distributions were used to describe categorical variables and mean, median, and ranges were used for quantitative variables. Demographic features were compared between the Gln+ and Gln- cohorts using a Chi-square test. The effects of Gln on acute and late radiation-induced esophageal toxicity, BMI change, WC, and PWC during treatment, and need for hospitalization and/or treatment breaks were comparatively analyzed. As these issues were previously addressed in our previous study, for this current study, the primary endpoints were determined to be differences in overall survival (OS), locoregional progression-free survival (LRPFS), and progression-free survival (PFS) between the two cohorts. OS, LRPFS, and PFS were calculated as the time between the first day of C-CRT and the date of death/last visit for OS, the date of local or regional relapse or the date of death/last visit for LRPFS, and any type of local/regional or distant progression of disease or the date of death/last visit for PFS. Survival analysis was performed by the Kaplan-Meier method and the survival curves of subsets were compared with two-sided log-rank tests. All tests were two-tailed, and a p-value <0.05 was considered significant.

## Results

Pretreatment characteristics of patients and disease are shown in Table 3. In general C-CRT was well tolerated in both cohorts. The unique acute toxicities experienced during the first week of Gln administration prior to initiation of C-CRT were mild nausea in 10 (17.9%) patients and vomiting in 4 (7.1%) patients, both of which were successfully treated with metoclopramide. During the course of C-CRT there was no grade ≥3 nausea or vomiting, and the rates of grade 1–2 nausea and vomiting were 32.1% and 19.6% respectively for Gln+ cohorts and 29.2% and 16.7% for Gln- cohorts (p>0.05 for each).

**Table 3:** Pretreatment patient and disease characteristics

**Characteristic**	**All**	**Glutamine (+)**	**Glutamine (−)**	**P-value**
	**(N=104)**	**(N=56)**	**(N=48)**	
**Age (years)**				
Median (Range)	57.6 (33–69)	58.7 (41–69)	56.5 (33–69)	0.41
**Gender (N; %)**				
Male	67 (64.4)	35 (62.5)	32 (66.7)	0.62
Female	37 (35.6)	21(37.5)	16 (33.3)	
**Histology (N; %)**				
Squamous cell	64 (61.5)	34 (60.7)	30 (62.5)	0.81
Adeno	40 (38.5)	22 (39.3)	18 (37.5)	
**KPS (N; %)**				
90 – 100	58 (55.8)	30 (53.6)	28 (58.3)	0.76
70 - 80	46 (44.2)	26 (46.4)	20 (41.7)	
**TN-stage (N; %)**				
T1N3	7 (6,7)	4 (7,1)	3 (6,3)	0.38
T2N3	13 (12.5)	6 (10.7)	7 (14.5)	
T3N3	17 (16.3)	10 (17.8)	7 (14.5)	
T4N0	11 (10.6)	6 (10.7)	5 (10.4)	
T4N1	16 (15.4)	8 (14.3)	8 (16.7)	
T4N2	18 (17.3)	10 (17.9)	8 (16.7)	
T4N3	22 (21.2)	12 (21.5)	10 (20.9)	
**T-stage (N; %)**				
1	7 (6.7)	4 (7.1)	3 (6.3)	0.33
2	13 (12.5)	6 (10.7)	7 (14.5)	
3	17 (16.3)	10 (17.8)	7 (14.5)	
4	67 (64.5)	36 (64.4)	31 (64. 7)	
**N-stage (N; %)**				
0	11 (10.6)	6 (10.7)	5 (10.4)	0.58
1	16 (15.4)	8 (14.3)	8 (16.7)	
2	18 (17.3)	10 (17.9)	8 (16.7)	
3	59 (56.7)	32 (57.1)	25 (58.1)	
**Bulk of T (N; %)**				
≤ 3.0 cm	8 (7.7)	3 (5.4)	5 (10.4)	0.42
3.01 - 5.0 cm	15 (14.4)	8 (14.3)	7 (14.6)	
5.01 - 7.0 cm	43 (41.3)	24 (42.9)	19 (39.6)	
> 7.0 cm	38 (36.6)	21 (37.4)	17 (35.4)	
**Bulk of largest N (N; %)**				
≤ 2.0 cm	58 (55.8)	30 (53.7)	28 (58.3)	0.22
> 2.0 cm	46 (44.2)	26 (46.3)	20 (41.7)	
**Chemotherapy**				
Platin – docetaxel	46 (44.2)	24 (42.9)	22 (45.8)	0.79
Platin - vinorelbine	58 (55.8)	32 (57.1)	26 (54.2)	
**Weight (kg)**				
Median (range)	66.3 (50.5-87.6)	65.9 (50.5-86.8)	67.2 (54.6-87.6)	0.37
**BMI (kg/m2)**				
Median (range)	22.1 (18.4-27.8)	21.8 (18.4-27.6)	22.3 (18.8-27.8)	0.91

**Abbreviations:***BMI*: Body mass index; *KPS*: Karnofsky performance score; *N*: Node; *T*: Tumor.

No grade 4–5 ARIE was reported in Gln+ or Gln- cohorts. As shown in Table 4, comparative analysis revealed a significantly lower incidence of grade 3 ARIE in the Gln+ cohort than in the Gln- cohort (7.2% vs. 16.7%; p=0.02). Diagnosis of maximum grade ARIE was delayed by 8 days with the use of Gln (24.5 vs. 16.4 days, p=0.001). Unplanned treatment delays, either by frequency or time, were also significantly lower in the Gln+ cohort. Hospitalization was needed in 5 (4.8%) patients: 3 (6.3%) in the Gln- cohort and 2 (3.6%) in the Gln+ cohort (p=0.14), and all patients were able to complete C-CRT with appropriate treatment and supportive measures as indicated. Over the long-term, no grade 4/5 late esophageal toxicity (LET) was reported in either cohort. The incidence of grade 2/3 LET was higher in the Gln- cohort than the Gln+ cohort (12.6% vs. 3.6%), approaching statistical significance (p=0.06).

**Table 4:** Treatment outcomes

**Characteristic**	**Glutamine (+)**	**Glutamine (−)**	**P-value**
	**(N=56)**	**(N=48)**	
**Maximum grade ARIE (N; %)**			
0–1	40 (71.4)	21 (43.7)	0.02
2	37.0	34.2	
3	27.8	22.8	
4 - 5	0 (0)	0 (0)	
**Grade 2–3 ARIE onset (days)**			
Median	24.5	16.4	0.001
Range	(17 – 32)	(9–23)	
**Treatment delay (N; %)**	4 (7.1)	10 (20.8%)	0.04
**Hospitalization**	2 (3.6)	3 (6.3)	0.14
**Weight change (N; %)**			
No change or gain	31 (55.4)	13 (27.1)	0.002
Loss	25 (44.6)	35 (72.9)	
**Weight change (kg)**			
Median	2.6	−3.3	< 0.001
Range	(−3.1 to 7.6)	(−9.7 to 2.3)	
**Weight change (%)**			
Median	3.94	−4.91	< 0.001
Range	(−4.7 to 11.5)	(−14.4 to 3.4)	
**LET (maximum grade)**			
2	2 (3.6)	3 (6.3)	0.06
3	0 (0)	3 (6.3)	
4 - 5	0 (0)	0 (0)	

**Abbreviations:***ARIE*: Acute radiation-induced esophagitis; *LET*: Late esophageal toxicity.

Although all other supportive measures were similar between cohorts, Gln- patients experienced significant weight loss, negative PWC, and negative BMI change, whereas Gln+ patients maintained or gained weight at the end of the C-CRT course, as reflected in the PWC and BMI measurements (Table 4).

At a median follow-up of 24.2 months (range 5.2-37.8), 45 patients (36.9%) were alive [23 Gln+ (41.1%) and 22 Gln- (45.8%)], and 17 (16.3%) of these were free of disease progression [10 Gln+ (17.9%) and 7 Gln- (14.6%)]. Analysis of response rates according to EORTC-1999 criteria and relapse patterns revealed no significant difference between the two cohorts (p>0.05; Table 5). Partial response and distant relapses were the most common response and relapse patterns in both Gln+ and Gln- cohorts.

**Table 5:** Locoregional response and relapse characteristics for patients with and without glutamine supplementation

**Characteristic**	**All**	**Glutamine (+)**	**Glutamine (−)**	**P-value**
	**(N=104)**	**(N=56)**	**(N=48)**	
**Locoregional response (N; %)**				
Complete	15 (14.4)	8 (14.3)	7 (14.5)	0.79
Partial	34 (32.7)	18 (32.1)	16 (33.4)	0.62
Stable	28 (26.9)	16 (28.6)	12 (25.0)	0.31
Progression	27 (26.0)	14 (25.0)	13 (27.1)	0.43
**Relapse pattern (N; %)**				
None	19 (18.3)	10 (17.9)	9 (18.8)	0.42
Locoregional	20 (19.2)	10 (17.9)	10 (20.8)	0.59
Distant	46 (44.2)	25 (44.6)	21 (43.7)	0.30
Locoregional + distant	19 (18.3)	11 (19.6)	8 (16.7)	0.24

**Figure 1:**
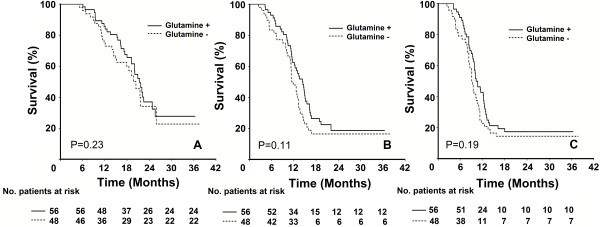
**Comparative survival analyses between Gln+ and Gln- cohorts.A**: Overall survival (OS); **B**: Progression-free Survival (PFS); **C**: Local Regional Progression-free Survival (LRPFS). Solid line: Gln+; Dashed line: Gln-.

Median OS, LRPFS, and PFS for the entire population were 20.9 (95% CI: 19.5-22.3), 12.7 (95% CI: 11.5-13.5), and 9.7 months (95% CI: 9.0-10.4), respectively. Corresponding 2-and 3-year survival estimates were 34.9% and 25.4% for OS; 16.8% and 16.8% for LRPFS; and 16.1% and 16.1% for PFS, respectively. As shown in Figure 1 and Table 6, intergroup comparisons between Gln+ and Gln- cohorts revealed no statistically significant differences in median 2- and 3-year OS, LRPFS, and PFS.

**Table 6:** Survival estimates according to prophylactic glutamine use

**Survival**	**Glutamine (+)**	**Glutamine (−)**	**P-value**
	**(N=56)**	**(N=48)**	
**Overall**			
Median (months)	21.4	20.4	0.23
2-year (%)	37.0	34.2	
3-year (%)	27.8	22.8	
**Locoregional progression free**			
Median (months)	11.3	14.2	0.11
2-year (%)	18.7	16.4	
3-year (%)	18.7	16.4	
**Progression free**			
Median (months)	10.2	9.0	0.19
2-year (%)	17.5	14.6	
3-year (%)	17.5	14.6	

## Discussion

Despite the potential unpredictable disadvantages of any retrospective analysis, in the dose and schedule utilized here, present results showed that besides being beneficial in prevention of weight loss, unplanned treatment delays, severity and incidence of acute and late RIE, co-administration of Gln during C-CRT has no detectable negative impact on tumor control and survival outcomes in patients with stage IIIB NSCLC.

One strategy to reduce radiation-induced normal tissue toxicity is the use of protective pharmacologic agents shortly before and/or during the course of RT/C-CRT. Recent preclinical studies revealed that Gln, the primary fuel of enterocytes and lymphocytes, not only plays a crucial role in maintaining gut integrity and cellular immunity [3,21-24] but also protects against acute and late radiation-induced injury by inhibiting bacterial translocation and stimulating production of the antioxidant GSH [25-29]. Clinically, oral Gln reduces the incidence and severity of RT- and/or chemotherapy-induced mucosal injury at various tumor sites, including the esophagus in NSCLC [9,10,30-32]. Similarly, our current findings showed that Gln prophylaxis was associated with significantly reduced rates of grade 3 ARIE incidence (7.2% vs.16.8%; p=0.02), and delayed onset of maximum grade ARIE (24.5 vs. 16.4 days; p=0.001) with no add on toxicity.

Considering its selective protective function in normal non-cancerous tissues, ease of use, and mild and easily manageable toxicity profile, Gln appears to be an ideal radioprotector. However, there are concerns that Gln may protect tumor cells, or even promote tumor growth, when used in conjunction with anticancer treatment [13-15]. To our knowledge, no previous clinical study has specifically addressed the influence of Gln on tumor control and survival outcomes when administered during C-CRT in NSCLC patients, and the results of studies on other tumor sites are conflicting [17,33-36]. Therefore, this is the first report of the effects of Gln on survival outcomes, and indirectly, tumor growth kinetics of LA-NSCLC in the era of RT/C-CRT.

Although the fact that human tumors exhibit a 5- to 10-fold faster rate of Gln consumption than normal healthy tissues [37-39] might suggest that supplemental Gln would promote growth of tumor calls [13-15], Gln did not stimulate tumor growth or negatively affect the outcome of any type of anti-tumor treatment in this study and previously published reports [8,21,22,40,41]. In experimental studies, Gln supplementation has repeatedly been shown to replete Gln stores in muscle with no promotion of tumor growth which was proved by absence of any notable increment in tumor DNA content [8,21,22,40]. Furthermore, Fahr and colleagues [41] demonstrated that Gln gavage and pair-fed food combination was associated with a 30% increment in natural killer (NK) cell activity and a 40% reduction in tumor growth. Use of Gln in conjunction with chemotherapy and/or RT has been investigated in only a limited number of clinical trials. In a large randomized, double-blind, placebo-controlled study [33], oral Gln supplementation was associated with significantly reduced mouth pain and, more importantly, improved survival rates at 28 days in 193 patients undergoing autologous or allogeneic bone marrow transplant. In a similar patient group, Schloerb and Skikne [34] reported significantly improved long-term survival with parenteral Gln supplementation. In the setting of RT or C-CRT, the few published studies concentrated on the radioprotective actions of Gln without considering its potential impact on tumor growth and survival outcomes [9,10,32,42,43]. Consistent with recently reported C-CRT studies without Gln [44-49], the similar PFS, LRPFS, and OS for Gln+ and Gln- cohorts observed in the current study demonstrated no association between tumor growth stimulation and high-dose Gln administered during C-CRT of LA-NSCLC patients.

If Gln is not provided exogenously tumor cells can successfully manipulate host metabolism to cover their needs, therefore artificial depletion of Gln cannot stop, or even retard, tumor growth. In fact, Gln-deprivation increases tumor cell survival through the induction of pro-angiogenic, pro-metastatic, pro-inflammatory, and tumor motility factors such as VEGF, IL-8, and NF-_K_B [4]. Moreover, lack of supplementary Gln can lead to serious Gln depletion, which is closely associated with impaired physiological functions such as disturbances in mucosal integrity, immune competence, maintenance of normal tissue GSH levels, and inhibition of bacterial translocation, resulting in serious medical complications. Therefore, exogenous Gln utilized here appears to improve the general metabolic condition and host defense mechanisms, and decrease the C-CRT-induced toxicity and related detrimental effects on quality of life measures and clinical outcomes.

One important consequence of dose-limiting acute toxicities of RT, and particularly C-CRT, in LA-NSCLC patients is the need for unplanned treatment breaks, which mandates reductions in doses of chemotherapy/RT and/or prolongs the overall treatment time with the potential to induce accelerated tumor repopulation [50]. Overall, any prolongation in treatment course is strongly associated with significantly reduced efficacy of C-CRT and therefore reduced rates of locoregional control and survival [51]. Our study showed that Gln significantly reduced the incidence and delayed the onset of grade ≥3 ARIE, reduced the need for unplanned treatment breaks, and reduced hospitalization. Although our study failed to show a significant survival advantage, further studies with larger study cohorts and sufficient statistical power to detect a moderate survival advantage are warranted.

The present study has several limitations. First, as for any retrospective study, unpredictable biases may have influenced our results. Second, heterogeneity due to inclusion of both adeno- and squamous cell cancer histologies, together with the limited cohort size, probably decreased the statistical power to identify a subgroup that may have benefited from Gln supplementation in terms of tumor control and survival outcomes. Third, although not significant statistically, the survival rates of the Gln+ cohort were higher than those of the Gln- cohort at all time points, suggesting that patients who received Gln supplementation tended to do better than those who did not. This may be partly associated with the small sample size and relatively short follow-up period and should be further addressed in larger studies with a longer follow-up period. Finally, although our institutional policy mandates arrangement of nutritional status of patients prior to treatment, nutritional differences are strongly associated with general feeding behaviors and socioeconomic status and cannot easily be controlled between the groups which may also affected our results.

## Conclusion

Our analysis showed that supplemental use of Gln during C-CRT has no detectable negative impact on tumor control and survival outcomes in patients with Stage IIIB NSCLC, but rather might prevent weight loss and unplanned treatment delays and reduce the severity and incidence of acute and late RIE. However, prospective randomized studies with larger cohorts and statistical power or comprehensive meta-analyses are warranted to conclude more relevantly on this continuously discussed specific issue of oncology.

## Competing interests

We have no personal or financial conflict of interest and have not entered into any agreement that could interfere with our access to the data on the research, or upon our ability to analyze the data independently, to prepare manuscripts, and to publish them.

## Authors’ contributions

Study conception and design: ET. Provision of study materials or patients: ET, ST, CP. Collection and assembly of data: ET, CP, BP. Data analysis and interpretation: ET, CP. Manuscript writing: ET, CP. Final approval of manuscript: ET, CP, ST, BP.

## Pre-publication history

The pre-publication history for this paper can be accessed here:

http://www.biomedcentral.com/1471-2407/12/502/prepub
